# Fractalkine and Other Chemokines in Primary Biliary Cirrhosis

**DOI:** 10.1155/2012/102839

**Published:** 2011-08-09

**Authors:** Shinji Shimoda, Carlo Selmi, M. Eric Gershwin

**Affiliations:** ^1^Department of Medicine and Biosystemic Science, Kyushu University Graduate School of Medical Sciences, Kyushu University, Fukuoka 819-0395, Japan; ^2^Division of Internal Medicine, IRCCS Istituto Clinico Humanitas, via A. Manzoni 56, Rozzano, 20089 Milan, Italy; ^3^Division of Rheumatology, Allergy and Clinical Immunology, University of California at Davis, Davis, CA 95616, USA

## Abstract

Primary biliary cirrhosis (PBC) is characterized by the autoimmune injury of small intrahepatic bile duct. On this basis, it has been suggested that the targeted biliary epithelial cells (BEC) play an active role in the perpetuation of autoimmunity by attracting immune cells via chemokine secretion. To address this issue, we challenged BEC using multiple toll-like receptor (TLR) ligands as well as autologous liver infiltrating mononuclear cells (LMNC) with subsequent measurement of BEC phenotype and chemokine production and LMNC chemotaxis by quantifying specific chemokines, specially CX3CL1 (fractalkine). We submit the hypothesis that BEC are in fact the innocent victims of the autoimmune injury and that the adaptive immune response is critical in PBC.

## 1. Introduction

Primary biliary cirrhosis (PBC) is a chronic cholestatic liver disease recognized at histology as chronic nonsuppurative destructive cholangitis with an autoimmune pathogenesis supported by Th1 or Th17 cells producing IFN-*γ* or IL-17 [1, 2]. Several inflammatory cell populations, including T and B cells, are found around the affected intrahepatic bile ducts, and chemokines are believed to play a pivotal role for the infiltration of inflammatory cells [3]. 

A better understanding of the role of specific chemokines in liver injury is ancillary to understanding the molecular mechanisms regulating the autoimmunity process and is expected to unravel new strategies to treat PBC.

**Figure 1:**
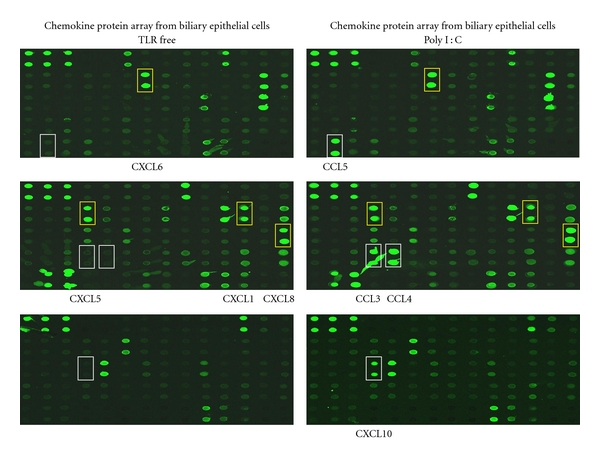
Chemokines produced by biliary epithelial cells under basal conditions or after stimulation with TLR3 ligand (poly I : C) for 48 hours. Cell-free culture supernatants were analyzed by a protein array kit to evaluate 174 different proteins simultaneously. Unstimulated cells produced detectable amounts of GRO-*α*/CXCL1, ENA-78/CXCL5, GCP-2/CXCL6, and IL-8/CXCL8, while poly I : C stimulation led to enhanced MIP-1*α*/CCL3, MIP-1*β*/CCL4, RANTES/CCL5, and IP-10/CXCL10.

The observed patterns of chemokine expression in normal and PBC liver are illustrated in Table 1 [4]. In our recent experiments, we cultured EpCAM-positive cells (i.e., biliary epithelial cells and BEC) isolated by immunobeads from explanted liver tissue and examined the production of chemokines by protein array following the stimulation by inflammatory cytokines or Toll-like receptor (TLR) ligands [5]. Our data illustrated that BEC produce proinflammatory chemokines such as CXCL1, CXCL5, CXCL6, and CXCL8 without any specific stimulation as shown in Figure 1. On the other hand, BEC challenged with a TLR3 ligand (poly I : C) manifest a Th1 shift and the production of CCL3, CCL4, CCL5, and CXCL10. Such production of Th1 chemokines was further prompted by the interaction between CD40 on BEC and CD154 on liver infiltrating lymphocytes. Taken altogether, the evidence support the observation that BEC induces a proinflammatory environment in the absence of innate immunity stimulation and induces Th1-sifted environment when such stimulation is present.

## 2. Fractalkine

Fractalkine is characterized as a type-1 transmembrane molecule with the chemokine domain tethered by a 241-amino acid glycosylated stalk, a 19-amino acid transmembrane region, and 37-amino acid intracellular tail [6]. The surface-expressed transmembrane fractalkine induces the firm adhesion of leukocytes expressing its receptor CX3CR1. After shedding by the disintegrins and metalloproteinases (ADAM) 10 and 17, fractalkine also acts as a soluble leukocyte chemoattractant. Transmembrane fractalkine expressed on both endothelial and epithelial cells induces leukocyte transmigration [7]. Fractalkine is upregulated by inflammation cytokines such as TNF-*α* or IFN-*γ*, it has been proposed to contribute to inflammatory diseases by promoting the transmigration of CX3CR1-expressing cells to inflamed tissues in Crohn disease [8], rheumatoid arthritis, atherosclerosis [9], systemic lupus erythematosus [10], and most recently PBC [5]. CX3CR1 is expressed on natural killer cells, monocytes, macrophages, mucosal dendritic cells, CD8^+^ T cells, and a subset of effector-memory CD4^+^ T cells [11, 12]. Human Th1 cells express high levels of CX3CR1 mRNA, different from polarized Th2 cells [13, 14]. Fractalkine is expressed in limited amounts in the normal human liver, particularly near branches of the hepatic artery and in small bile ducts located at the interface between the portal tract and the hepatic lobule. In the case of acute or chronic viral hepatitis, fractalkine is detected in the areas of necrosis and inflammatory infiltration and also at the interface between the expanded portal tract and the regenerating nodule. Regenerating epithelial cells of the ductular reaction are also positive for fractalkine [15]. In kidney allograft transplantation, fractalkine is expressed in renal tubular epithelial cells, and the expression is upregulated by TNF-*α*, the recognized key proinflammatory cytokine in acute rejection [16]. The CD4^+^ and CD8^+^ T cells expressing CX3CR1 predominantly produce IFN-*γ* and TNF-*α*, and these T cells infiltrate the synovium in patients with rheumatoid arthritis [17]. In inflammatory bowel disease (IBD), intestinal microvascular endothelial cells produce high amounts of fractalkine, and IBD mucosa as well as periphery contained significantly more CX3CR1+ cells than control. Fractalkine is a major contributor to T- and monocytic-cell adhesion to endothelial cells [18]. In HCV infection, CX3CR1 is susceptible gene for hepatic fibrosis [19]. In mice models, it is unclear whether CX3CR1 positive cells are protective or trigger disease [20–25].

## 3. Fractalkine and PBC

**Figure 2:**
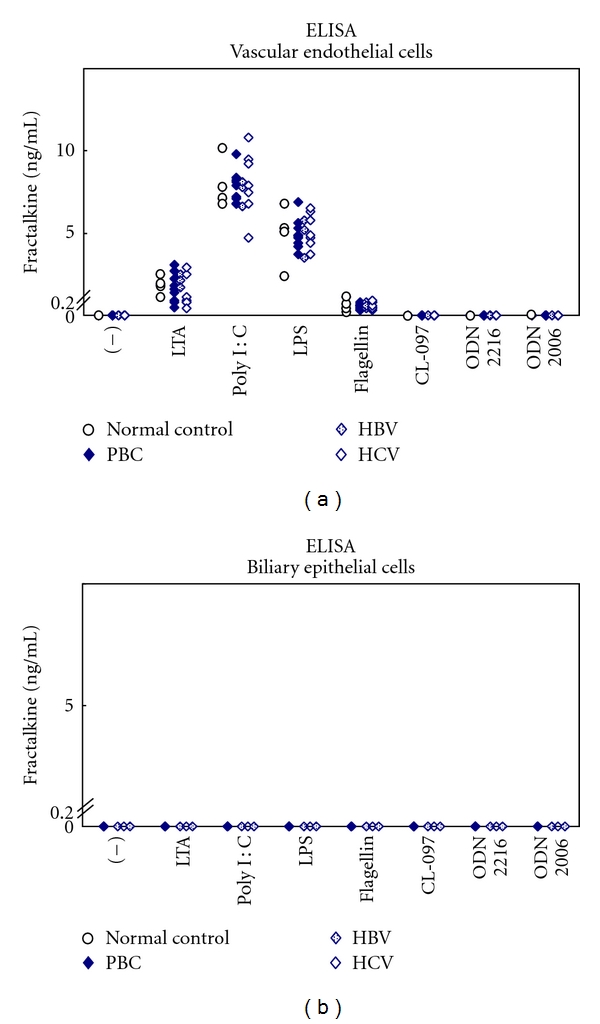
(a) Fractalkine production from endothelial cells from PBC and control (chronic hepatitis B and C) livers exposed to TLR ligands. Endothelial cells produced fractalkine with LTA, poly I : C, LPS, and flagellin with no significant differences observed between patients and control livers. (b) BEC did not produce fractalkine with any additional TLR ligand.

**Figure 3:**
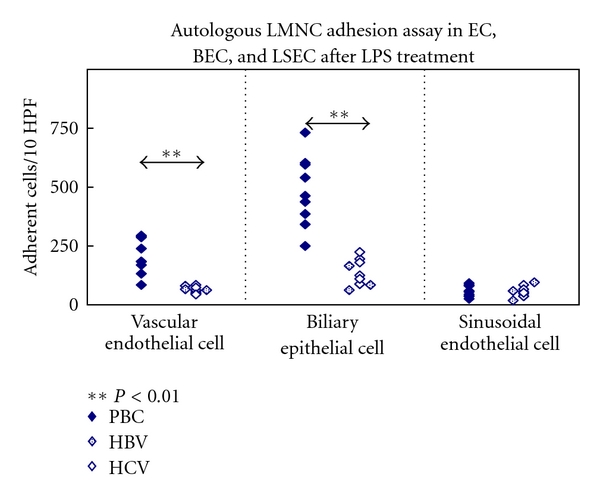
Autologous liver mononuclear cells adhesion assay using endothelial cells and BEC after stimulation with TLR4 ligand (LPS). Adherent liver mononuclear cells were stained and counted in ten random high-power microscopy fields. Liver mononuclear cells from PBC livers adhered in greater numbers than did liver mononuclear cells from controls using either endothelial cells or biliary epithelial cells, whereas liver mononuclear cells adhered only minimally to liver sinusoidal endothelial cells in all instances. Other TLR ligands did not accelerate liver mononuclear cells adhesion with neither endothelial cells nor biliary epithelial cells (data not shown).

**Figure 4:**
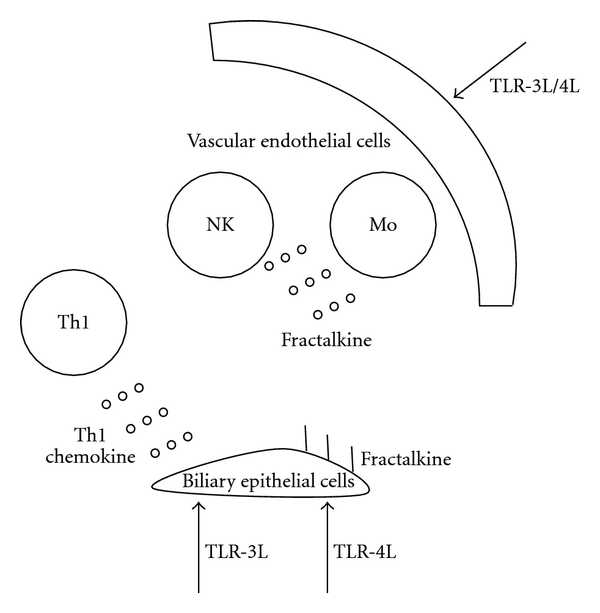
The proposed role of fractalkine is illustrated. TLR3 or TLR4 ligands stimulate vascular endothelial cells to produce fractalkine as chemokine, then fractalkine attracts CX3CR1 positive monocytes or NK cells. Subsequently, TLR4 ligand stimulated BEC produce fractalkine as cell adhesion molecule, then fractalkine recruit CX3CR1 positive cells around PBC target cells. This starts the chronic nonsuppurative destructive cholangitis and perpetuates the autoimmune pathogenesis of disease. Finally, TLR3 ligand stimulated biliary epithelial cells produce Th1 chemokines, and these chemokines are considered to contribute this autoimmune mechanism.

Fractalkine is peripherally expressed dominantly in patients with PBC, and is upregulated in BEC of the PBC liver. CX3CR1 is expressed on infiltrating lymphocytes in the portal tracts and on intraepithelial T cells of injured bile ducts [26]. BEC manifesting senescent features in damaged small bile ducts also overexpress fractalkine [27]. As previously introduced, in our recent work, we separated BEC as EpCAM positive and endothelial cells as CD31 positive by immunobeads and evaluated the production of fractalkine as chemokine by ELISA.  Figure 2(a) illustrates the elevated production of fractalkine by endothelial cells challenged with TLR3 ligand (poly I : C) or TLR4 ligand (LPS). Conversely, BEC did not produce fractalkine with any other TLR ligand stimulation (Figure 2(b)), and this was not reversed with the addition of established inflammatory cytokines such as TNF-*α* or IFN-*γ*. Further, we investigated the production of fractalkine following the interaction between BEC or endothelial cells and liver infiltrating lymphocytes. As shown in Figure 3, mononuclear cells adhered with higher affinity to BEC compared to endothelial cells in the TLR4 ligand (LPS) stimulation, and this adherence was increased more in PBC than in other control diseases [5]. Fractalkine works to modulate inflammation in the BEC of PBC, thus suggesting that novel therapies to block fractalkine induced environment may prove beneficial. Based on our data, we propose a working model on the role of fractalkine as chemokine or cell adhesion molecule by vascular endothelial cells and BEC, summarized in Figure 4. First, fractalkine as chemokine from vascular endothelial cells stimulated via TLR3 or TLR4 induce CX3CR1 positive monocytes or NK cells. Second, fractalkine as cell adhesion molecule from TLR4-stimulated BEC recruit CX3CR1 positive cells around target cells. This mechanism may trigger the onset of chronic nonsuppurative destructive cholangitis and autoimmune mechanism perpetuating the cholangitis. We further submit that Th1 chemokines produced by BEC stimulated from TLR3 are important contributors to the autoimmune mechanism.

## Figures and Tables

**Table 1:** Chemokine expression patterns in the portal tract, sinusoidal endothelium, and bile duct of normal and PBC liver.

Chemokine	Portal vein		Sinusoidal EC		Bile duct	
	Normal	PBC	Normal	PBC	Normal	PBC
CXCL9	±	+(?)	±	ND	−	+
CXCL10	±	+(?)	±	ND	−	+
CXCL11	±	ND	+	ND	ND	ND
CXCL12	−	−	−	−	+	++
CXCL16	+	+	+	+	+	++
CCL25	−	−	−	−	−	−
CCL28	−	+	−	−	−	++
